# Supplementary material for: Association between restless legs syndrome and sleep quality in Peruvian medical students
Source: PLoS One. 2025 Mar 25;20(3):e0320008. doi: 10.1371/journal.pone.0320008 (PMC11936163; doi:10.1371/journal.pone.0320008)
Supplement: S1 File — (DOCX) [file pone.0320008.s001.docx]

**Supplementary Materials S1: Study survey**

Edad

Sexo

(0) Masculino

(1) Femenino

Estado civil

(1) Soltero - sin pareja

(2) Soltero - con pareja

(3) Casado

(4) Conviviente

(5) Divorciado o separado

¿De qué departamento procede?

(1) Amazonas

(2) Ancash

(3) Apurímac

(4) Arequipa

(5) Ayacucho

(6) Cajamarca

(7) Callao

(8) Cusco

(9) Huancavelica

(10) Huánuco

(11) Ica

(12) Junín

(13) La Libertad

(14) Lambayeque

(15) Lima

(16) Loreto

(17) Madre de Dios

(18) Moquegua

(19) Pasco

(20) Piura

(21) Puno

(22) San Martín

¿Cómo sustenta sus gastos diarios?

(1) Trabaja

(2) Recibe dinero de sus familiares

(3) Trabaja y recibe dinero de sus familiares

¿En qué tipo de universidad estudia?

(1) Nacional

(2) Privada

¿En qué universidad estudia?

¿Qué año cursa?

(1) Primer año

(2) Segundo año

(3) Tercer año

(4) Cuarto año

(5) Quinto año

(6) Sexto año

¿Practica algún deporte al menos una vez en la semana?

(1) Sí

(2) No

¿Realiza actividad física 30 minutos o más por día al menos 5 veces a la semana?

(1) Sí

(2) No

**Índice de calidad de sueño**

1. Durante el último mes, ¿Cuál ha sido, normalmente, su hora de acostarse? APUNTE SU HORA HABITUAL DE ACOSTARSE:

2. ¿Cuánto tiempo habrá tardado en dormirse, normalmente, las noches del último mes? APUNTE EL TIEMPO EN MINUTOS:

3. Durante el último mes, ¿a qué hora se ha levantado habitualmente por la mañana? APUNTE SU HORA HABITUAL DE LEVANTARSE:

4. ¿Cuántas horas calcula que habrá dormido verdaderamente cada noche durante el último mes? (El tiempo puede ser diferente al que usted permanezca en la cama) APUNTE LAS HORAS QUE CREA HABER DORMIDO:

Para cada una de las siguientes preguntas, elija la respuesta que más se ajuste a su caso. Intente contestas a todas las preguntas.

5. Durante el último mes, cuántas veces ha tenido problemas para dormir a causa de:

a) No poder conciliar el sueño en la primera media hora:

Ninguna vez en el último mes

Menos de una vez a la semana

Una o dos veces a la semana

Tres o más veces a la semana

b) Despertarse durante la noche o de madrugada:

Ninguna vez en el último mes

Menos de una vez a la semana

Una o dos veces a la semana

Tres o más veces a la semana

c) Tener que levantarse para ir al servicio:

Ninguna vez en el último mes

Menos de una vez a la semana

Una o dos veces a la semana

Tres o más veces a la semana

d) No poder respirar bien:

Ninguna vez en el último mes

Menos de una vez a la semana

Una o dos veces a la semana

Tres o más veces a la semana

e) Toser o roncar ruidosamente:

Ninguna vez en el último mes

Menos de una vez a la semana

Una o dos veces a la semana

Tres o más veces a la semana

f) Sentir frío:

Ninguna vez en el último mes

Menos de una vez a la semana

Una o dos veces a la semana

Tres o más veces a la semana

g) Sentir demasiado calor:

Ninguna vez en el último mes

Menos de una vez a la semana

Una o dos veces a la semana

Tres o más veces a la semana

h) Tener pesadillas o «malos sueños»:

Ninguna vez en el último mes

Menos de una vez a la semana

Una o dos veces a la semana

Tres o más veces a la semana

i) Sufrir dolores:

Ninguna vez en el último mes

Menos de una vez a la semana

Una o dos veces a la semana

Tres o más veces a la semana

j) Otras razones (por favor, descríbalas a continuación):

Ninguna vez en el último mes

Menos de una vez a la semana

Una o dos veces a la semana

Tres o más veces a la semana

6. Durante el último mes, ¿cómo valoraría, en conjunto, la calidad de su sueño?

Bastante buena

Buena

Mala

Bastante mala

7. Durante el último mes, ¿cuántas veces habrá tomado medicinas (por su cuenta o recetadas por el médico) para dormir?

Ninguna vez en el último mes

Menos de una vez a la semana

Una o dos veces a la semana

Tres o más veces a la semana

8. Durante el último mes, ¿cuántas veces ha sentido somnolencia mientras conducía, comía, o desarrollaba alguna otra actividad?

Ninguna vez en el último mes

Menos de una vez a la semana

Una o dos veces a la semana

Tres o más veces a la semana

9. Durante el último mes, ¿ha representado para Vd,

mucho problema el «tener ánimos» para realizar

alguna de las actividades detalladas en la pregunta

anterior?

10. ¿Duerme usted solo o acompañado?

Solo

Con alguien en otra habitación

En la misma habitación, pero en otra cama

En la misma cama

POR FAVOR, SÓLO CONTESTE A LAS SIGUIENTES PREGUNTAS EN EL CASO DE QUE DUERMA ACOMPAÑADO

Si tiene pareja o compañero de habitación, pregúntele si durante el último mes usted ha tenido:

a) Ronquidos ruidosos.

Ninguna vez en el último mes

Menos de una vez a la semana

Una o dos veces a la semana

Tres o más veces a la semana

b) Grandes pausas entre respiraciones mientras duerme.

Ninguna vez en el último mes

Menos de una vez a la semana

Una o dos veces a la semana

Tres o más veces a la semana

e) Sacudidas o espasmos de piernas mientras duerme.

Ninguna vez en el último mes

Menos de una vez a la semana

Una o dos veces a la semana

Tres o más veces a la semana

d) Episodios de desorientación o confusión mientras duerme.

Ninguna vez en el último mes

Menos de una vez a la semana

Una o dos veces a la semana

Tres o más veces a la semana

e) Otros inconvenientes mientras Vd. duerme (Por favor, descríbalos a continuación)

Ninguna vez en el último mes

Menos de una vez a la semana

Una o dos veces a la semana

Tres o más veces a la semana

**Síndrome de piernas inquietas**

¿Tiene sensación de disconfort o molestias en sus piernas, combinadas con la urgencia o necesidad de moverlas?

(1) Sí

(2) No

Estas molestias, ¿ocurren solo en reposo y mejoran con el movimiento?

(1) Sí

(2) No

¿Son peores en la tarde noche que en la mañana?

(1) Sí

(2) No

**Síntomas de ansiedad**

Responde o marca con un aspa (X) la casilla con la letra correspondiente:

Se asusta súbitamente sin motivo

(1) Nada

(2) Un poco

(3) Bastante

(4) Mucho

Siente que tiene miedo

(1) Nada

(2) Un poco

(3) Bastante

(4) Mucho

Se desmaya, se marea o se siente débil

(1) Nada

(2) Un poco

(3) Bastante

(4) Mucho

Siente nerviosismo o inquietud en su interior

(1) Nada

(2) Un poco

(3) Bastante

(4) Mucho

Su corazón late aceleradamente o más rápido de lo usual

(1) Nada

(2) Un poco

(3) Bastante

(4) Mucho

Temblor

(1) Nada

(2) Un poco

(3) Bastante

(4) Mucho

Se siente tenso/a o entrampado/a

(1) Nada

(2) Un poco

(3) Bastante

(4) Mucho

Dolores de cabeza

(1) Nada

(2) Un poco

(3) Bastante

(4) Mucho

Períodos de terror o pánico

(1) Nada

(2) Un poco

(3) Bastante

(4) Mucho

Siente inquietud o que no puede estar tranquilo

(1) Nada

(2) Un poco

(3) Bastante

(4) Mucho

**Síntomas de depresión**

Siente falta de fuerzas, lentitud

(1) Nada

(2) Un poco

(3) Bastante

(4) Mucho

Tiene sentimiento de culpa persistente

(1) Nada

(2) Un poco

(3) Bastante

(4) Mucho

Remordimientos

(1) Nada

(2) Un poco

(3) Bastante

(4) Mucho

Llora fácilmente

(1) Nada

(2) Un poco

(3) Bastante

(4) Mucho

Ha perdido el interés sexual o placer

(1) Nada

(2) Un poco

(3) Bastante

(4) Mucho

Falta de apetito

(1) Nada

(2) Un poco

(3) Bastante

(4) Mucho

Dificultad para dormir o continuar dormido

(1) Nada

(2) Un poco

(3) Bastante

(4) Mucho

Se siente desesperanzado/a sobre el futuro

(1) Nada

(2) Un poco

(3) Bastante

(4) Mucho

Se siente triste

(1) Nada

(2) Un poco

(3) Bastante

(4) Mucho

Se siente solo/a

(1) Nada

(2) Un poco

(3) Bastante

(4) Mucho

Ha pensado acabar con su vida

(1) Nada

(2) Un poco

(3) Bastante

(4) Mucho

Se siente atrapado o aprisionado

(1) Nada

(2) Un poco

(3) Bastante

(4) Mucho

Se preocupa excesivamente por las cosas

(1) Nada

(2) Un poco

(3) Bastante

(4) Mucho

Siente falta de interés por las cosas

(1) Nada

(2) Un poco

(3) Bastante

(4) Mucho

Siente que todo requiere demasiado esfuerzo

(1) Nada

(2) Un poco

(3) Bastante

(4) Mucho

Se siente inútil

(1) Nada

(2) Un poco

(3) Bastante

(4) Mucho

**Síntomas de nomofobia**

En las siguientes preguntas, marcar con un aspa (x) de acuerdo a su percepción, donde: 1 es "totalmente en desacuerdo" y 7 es "totalmente de acuerdo"

1. Me sentiría incómodo sin acceso constante a la información a través de mi smartphone.

1 = Totalmente en desacuerdo

7 = Totalmente de acuerdo

2. Me sentiría irritado si no pudiese buscar información en mi smartphone cuando quisiera.

1 = Totalmente en desacuerdo

7 = Totalmente de acuerdo

3. Estaría nervioso si no pudiese obtener noticias (p. ej., eventos, el tiempo, etc.) en mi smartphone.

1 = Totalmente en desacuerdo

7 = Totalmente de acuerdo

4. Estaría irritado si no pudiese usar mi smartphone y sus capacidades cuando quisiera.

1 = Totalmente en desacuerdo

7 = Totalmente de acuerdo

5. Me asustaría quedarme sin batería en mi smartphone.

1 = Totalmente en desacuerdo

7 = Totalmente de acuerdo

6. Entraría en pánico si me quedase sin saldo o sobrepasase mi límite mensual de datos.

1 = Totalmente en desacuerdo

7 = Totalmente de acuerdo

7. Si me quedase sin señal de cobertura de datos o no pudiera conectarme al wifi, comprobaría constantemente si tenzo señal o pudiera encontrar una red de wifi.

1 = Totalmente en desacuerdo

7 = Totalmente de acuerdo

8. Si no pudiese usar mi smartphone, tendría miedo a quedarme tirado en algún lugar.

1 = Totalmente en desacuerdo

7 = Totalmente de acuerdo

9. Si estuviese un rato sin poder comprobar mi smartphone, tendría deseos de poder mirarlo.

1 = Totalmente en desacuerdo

7 = Totalmente de acuerdo

10. Sentiría ansiedad si no pudiese comunicarme instantáneamente con mi familia y amigos.

1 = Totalmente en desacuerdo

7 = Totalmente de acuerdo

11. Estaría preocupado porque mi familia y amigos no podrían contactar conmigo.

1 = Totalmente en desacuerdo

7 = Totalmente de acuerdo

12. Estaría nervioso porque no podría recibir mensajes de texto y llamadas.

1 = Totalmente en desacuerdo

7 = Totalmente de acuerdo

13. Sentiría ansiedad porque no podría mantener el contacto con mi familia y amigos.

1 = Totalmente en desacuerdo

7 = Totalmente de acuerdo

14. Estaría nervioso porque no podría saber si alguien había intentado contactar conmigo.

1 = Totalmente en desacuerdo

7 = Totalmente de acuerdo

15. Sentiría ansiedad porque se hubiese roto mi contacto continuo con mi familia y amigos.

1 = Totalmente en desacuerdo

7 = Totalmente de acuerdo

16. Estaría nervioso porque estaría desconectado de mi identidad en línea.

1 = Totalmente en desacuerdo

7 = Totalmente de acuerdo

17. Estaría incómodo porque no podría estar al día con las redes sociales y redes en línea.

1 = Totalmente en desacuerdo

7 = Totalmente de acuerdo

18. Sentiría torpeza porque no podría comprobar mis notificaciones de actualizaciones de mis contactos v redes en línea.

1 = Totalmente en desacuerdo

7 = Totalmente de acuerdo

19. Sentiría ansiedad porque no podría comprobar mi correo electrónico.

1 = Totalmente en desacuerdo

7 = Totalmente de acuerdo

20. Me sentiría raro porque no sabría qué hacer.

1 = Totalmente en desacuerdo

7 = Totalmente de acuerdo
