# Supplementary material for: Association between restless legs syndrome and sleep quality in Peruvian medical students
Source: PLoS One. 2025 Mar 25;20(3):e0320008. doi: 10.1371/journal.pone.0320008 (PMC11936163; doi:10.1371/journal.pone.0320008)
Supplement: Attachment — Submitted filename: 1.First round of review_20240728_Yantai.pdf [file pone.0320008.s003.pdf]

I welcomed the opportunity to review this manuscript. The manuscript is very brief, but fascinating; and I could read it within my scope. This study evaluated the association between RLS and sleep quality in Peruvian medical students. Findings showed that students with RLS symptoms had a 5% higher prevalence of poor sleep quality than those without RLS. Moreover, the prevalence of poor sleep quality was very high (77.3%). It must be said that this study has considerable academic value and practical significance.

However, I believe this manuscript leaves room for improvement, which means that the manuscript needs a certain degree of revision (if we must define the degree, it can be said to be a ***major revision***). I will explain my opinions one by one below and request the author to respond to the review comments one by one and revise them carefully.

1. Title

The title clearly states the core— restless legs syndrome, sleep quality and in Peruvian medical students. However, critical time period is lacking. Most importantly, an important time period mentioned in this manuscript is the COVID-19 pandemic (lines 179–180). It has also been proven that the results of this study were greatly affected by this time period. Therefore, I think it is necessary to add "during the COVID-19 pandemic" become to "***Association between restless legs syndrome and sleep quality in Peruvian medical students during the COVID-19 pandemic***".

2. Abstract

The content is acceptable, there are only some formatting issues, which will be mentioned later.

3. Introduction

The introduction is very short, but it is quite informative. Generally speaking, there are the following two problems in this part:

- First, the review section of the Research Gap (lines 61–67) is insufficient. I think that simply saying "*Previous studies have yielded heterogeneous results and have important methodological limitations, including small sample sizes and absence of control for potential confounding variables.*" is not a robust gap. Here, I need you to review the situation of medical students in Peru (or at least South America) in detail and find out the real gap.
- Second, the research objectives should be followed by a detailed description of the **research hypotheses**.

4. Methods

4.1 In (lines 74):

If the content of **S1 Appendix** is not too long, it can be placed in the main text of the manuscript. Otherwise, not presenting this part will interfere with the reader's coherent reading flow.

4.2 In (line 91–125 of **Variables and measures**):

There are a series of issues here that need to be clarified or revised by the author:

- If I counted correctly, there are four established scales used here, namely Pittsburgh Sleep Quality Index (PSQI), International Restless Legs Syndrome Study Group (IRLSSG), Hopkins Symptom Checklist-25 (HSCL-25) and Nomophobia Questionnaire (NMP-Q). All the Cronbach's alpha coefficients in these four scales are not from your current research, but are cited and summarized from previous research. This practice can be called "reliability induction" in psychometrics, please refer to the two journal articles in brackets (https://doi.org/10.1177/0013164403261814; https://doi.org/10.1177/00131640021970682). This is an inappropriate approach. Therefore, I ask you to report the Cronbach's alpha and McDonald's omega coefficients for each dimension you are currently studying.
- I checked the journals you cited regarding the Spanish versions of the instruments. Yes, they all show that the psychometric properties of the Spanish versions of the instruments are adequate. However, you did not specify ***where you obtained the instruments***. Therefore, the journals you cited do not always have an appendix showing the official Spanish version of the instruments. I hope you explain how you obtained the instruments; these are requirements for Open Science. The following is an example for your reference:
"The SDQ is a brief screening instrument to measure behavioral difficulties, capturing the MHS of children and adolescents aged 4–16 years [?]. It comprises 25 items across five dimensions: Emotional Symptoms, Conduct Problems, Hyperactivity/Inattention, Peer Problems, and Prosocial Behavior, with five items in each dimension. Responses are categorized into a 3-point scale (0 = not true, 1 = somewhat true, 2 = certainly true), with a six-month recall period. A few items are reverse-scored. After recoding reverse-scored items, the total difficulties score (TDS) is obtained by summing up scores from all 20 items except those in the Prosocial Behavior. The possible range of TDS is 0–40, with cutoff values from the Shanghai norms classifying it as "Normal" (0–14), "Borderline" (15–17), and "Abnormal" (18–40) [?]. The Mandarin Chinese self-reported SDQ used in this study has been validated previously [??], and it was excerpted from the "Handbook of Commonly Used Psychological

Assessment Scales" published in the Chinese Mainland [?]. Its internal consistency of the TDS was appropriate, with Cronbach's alpha (α) coefficient of 0.936 and McDonald's omegas (ωt) coefficient of 0.943."

- I noticed that you only introduced the scoring criteria for Hopkins Symptom Checklist-25 (HSCL-25) and Nomophobia Questionnaire (NMP-Q), but did not introduce how to use them to divide the test subjects into Yes/No. However, your Table 1 does show the division, and the Note of Table 1 does not indicate the division criteria. Therefore, I think you need to add the division criteria when introducing the instruments.
- If you follow the Open Science requirements, your appendix should include the complete questionnaire (including four established scales) you used in your current study. Please consider adding it. If you cannot add it, please explain why.

5. Results

There are some formatting issues in the Results section, which require review and revision by the authors.

<u>5.1 In (Table 1–3):</u>

Please ensure that your manuscript contains only English, unless there are special cases. For example, if there is a Spanish "18 a 20" "21 a 23" "24 a más" in Table 1–3, please correct it to English.

<u>5.2 In (Table 2):</u>

- If I'm not mistaken, most of the data in Table 2 may use chi-square tests, but you didn't indicate it and only showed the *p*-values. Here, I need you to add the data of the corresponding test statistics. For example, if you use chi-square tests, report χ^2. In addition, report the effect size of each test.
- The bottom line of Table 2 is missing, please add it.

<u>5.3 In (entire "**5. Results**" section and entire manuscript, including references):</u>

You have used hyphen (-) in all Table, References and entire manuscript. Is this appropriate?
Please refer to the relevant information to distinguish the difference between these four symbols and the corresponding application:

- Hyphen: -
- Minus sign (negative sign): −
- EN dash: –
- EM dash: —

Please pay attention to distinguish the four horizontal bars:

Number	Symbol name	Symbol	Example	Insert a “proper” sign in MS Word
1	Hyphen	-	f-f	Enter **2010** first and then press **Alt** + **X** (i.e., the [-] between [0] and [=] on the computer keyboard)
2	Minus sign (negative sign)	−	f−f	Enter **2212** first and then press **Alt** + **X**
3	EN dash	–	f–f	Enter **2013** first and then press **Alt** + **X**
4	EM dash	—	f—f	Enter **2014** first and then press **Alt** + **X**

6. Discussion

In (lines 212–215):

“*On the other hand, this study is the first to evaluate the relationship between restless legs syndrome and sleep quality in medical students, using a multivariable model in which other confounding variables were adjusted and with a larger sample size than used in previous research.*”

This section obviously states the strengths of the research. However, it is not logical to put it here. You should put this section in the first paragraph of the discussion. Because the first paragraph of the discussion usually introduces the overall situation of the research and its uniqueness.

7. References

Accurate referencing is a key component of good academic practice and enhances the presentation of your work: it shows that your writing is based on knowledge and informed by appropriate academic reading. The Reference format of the PLOS ONE is the AMA Style. However, you did not present the literature strictly as required by the AMA Style. You can refer to the AMA Manual of Style (11th edition). Please pay attention to the following problems, and step by step, manually correct these issues. Please do not only use bibliographic management software (because if there is information missing in the original document, the data exported by the bibliographic management software will also be wrong). In addition, each of your references lacks doi. Please refer to the format in brackets (https://doi.org/10.1111/j.1524-4733.2007.00293.x) to add it and add it as a hyperlink.
