# Supplementary material for: Association between restless legs syndrome and sleep quality in Peruvian medical students
Source: PLoS One. 2025 Mar 25;20(3):e0320008. doi: 10.1371/journal.pone.0320008 (PMC11936163; doi:10.1371/journal.pone.0320008)
Supplement: Attachment — Submitted filename: Response to Reviewers.docx [file pone.0320008.s005.docx]

Dear editor,

Many thanks for your response to the submission of our paper

We have thoroughly revised the paper in response to the reviewers' comments. Our detailed responses to each of their points are provided below, with the original comments presented in italics.

**Journal requirements**

*When submitting your revision, we need you to address these additional requirements.*

*1. Please ensure that your manuscript meets PLOS ONE's style requirements, including those for file naming.*

Reply: We have reviewed and modified the manuscript to ensure it meets the style criteria.

*2. Please ensure that you have specified a) Did participants provide their written or verbal informed consent to participate in this study?*

*b) If consent was verbal, please explain i) why written consent was not obtained, ii) how you documented participant consent, and iii) whether the ethics committees/IRB approved this consent procedure.*

Reply: We have added that informed consent was obtained to participate in the questionnaire. The following was added: "In order for the students to participate in the survey, they first had to provide their informed consent."

*3. When completing the data availability statement of the submission form, you indicated that you will make your data available on acceptance. We strongly recommend all authors decide on a data sharing plan before acceptance, as the process can be lengthy and hold up publication timelines. Please note that, though access restrictions are acceptable now, your entire data will need to be made freely accessible if your manuscript is accepted for publication. This policy applies to all data except where public deposition would breach compliance with the protocol approved by your research ethics board. If you are unable to adhere to our open data policy, please kindly revise your statement to explain your reasoning and we will seek the editor's input on an exemption. Please be assured that, once you have provided your new statement, the assessment of your exemption will not hold up the peer review process.*

Reply: We have added the study's database as supplementary material (Supplementary material 2).

**Reviewer #1.**

*Overall, this study is good study expressing the relationship between sleep quality and restless leg syndrome.*

*In results section, authors have mentioned about the percentage of anxiety and depression symptoms but please mention why there was necessity to check these symptoms with sleep quality*

*Please resolve typing and grammar errors (example in line number 92, 95)*

Reply: As suggested, we have corrected the grammatical errors in those lines.

**Reviewer #2.**

*This neurological condition is novel as very few people talk about this. also it involves approx. 3000 students. so the research is good. I have 3 submissions:*

*1. The author has not provided the questionnaire of google form given to the students. many unreported/Asyptomatic cases may have been used in this study.*

Reply: We have added the questionnaire as supplementary material (Supplementary material 1).

*2. The authors should provide adequate justification for the use of the software in this study. They should explain why this particular software was chosen and how it aligns with the research objectives. By elaborating on the software's specific functionalities and how it supports the analysis of the data, the authors can enhance the validity and reliability of their findings. also the detailed statistics must be checked from a experienced biostatician.*

Reply: We recognize the validity of the observation. , but STATA was created in 1985, is frequently updated and is a well-established software widely recognized in health, economic, and social sciences, and supports conducting adjusted regressions. The analysis of this study was carried out by a psychologist trained in statistics.

*3. Control group is missing.*

Reply: We conducted a cross-sectional analytical study, in which the independent variable was restless legs syndrome (RLS), and the control group consisted of participants without RLS. Table 1 presents the total number of individuals with and without RLS, and Table 3 identifies the “reference group” (i.e., control group) as individuals without RLS.

**Reviewer #3.**

*Copaja-Corzo et al conducted a study to evaluate the association between restless legs syndrome and sleep quality in Peruvian medical students, using cross-sectional survey data. And I would like to provide my feedback on the manuscript and recommend revisions for its improvement.*

*1. Inconsistency: several inconsistent descriptions appeared, and I would highly suggest the authors to examine carefully throughout. Examples are as follows:*

** In the abstract: “22.8% had poor sleep quality” was used, however, in the main body and the result table, the correct number should be “77.3%”*

Reply: We agree. In the abstract, we have changed “22.8% had poor sleep quality” to “77.3% had poor sleep quality”. In the Results section, we have changed “Additionally, 15.3% presented RLS symptoms, while 22.8% (95% CI: 25.9% - 29.1%) had poor sleep quality” to “Additionally, 15.3% presented RLS symptoms, while 77.3% had poor sleep quality”.

** PR (Prevalence ratio) was misused with “RP”*

Reply: We have corrected the grammatical error.

** The data sample was either “3139” (used in main body) or “3129” (used in the abstract)?*

Reply: In the abstract, we have changed “3129” to “3139”.

*2. Data collection: in the discussion, it was mentioned that the data was collected during the pandemic. It’s suggested to elaborate more on this in the Methods section.*

Reply: We have added more details about the data collection in the Methods section:

“The primary study collected data in two stages between June 2020 and March 2021. This was done virtually through social media platforms (Facebook, WhatsApp) because universities in Peru held online classes during the pandemic to minimize the risk of contagion.

The data collection occurred in two stages. In the first stage, a Facebook page was created to promote the research project. An open invitation was extended to all medical students in Peru through posters distributed in private Facebook groups for medical students, as well as through paid Facebook ads specifically targeting medical students in Peru.

In the second stage, 23 students from various medical schools were recruited through networks within the Scientific Society of Medical Students of Peru. The recruited students attended a 45-minute virtual meeting, where they were trained to contact students from their respective universities. As an incentive, all students who completed the survey were given free access to a Google Drive folder with a collection of resources from medical courses”.

*3. Discussion: “Although an association was found in our study, the difference between those who present RLS and those who do not is very small (5%).” This conclusion may not be applicable, since the reference study used odds vs. prevalence ratio was used in this study.*

Reply: We changed the sentence and specified that we are referring to the prevalence in Discussion section: “although an association was found in our study, the difference in prevalence between those who present RLS and those who do not is very small (5%)”.

**Reviewer #4.**

*Dear Editor,*

*I have reviewed the manuscript entitled "Association between restless legs syndrome and sleep quality in Peruvian medical students." Overall, the article is well written and the methodology is convincing. However, there are a few areas where additional clarification and detail would enhance the manuscript's clarity and robustness.*

*Firstly, in the Methods section, the authors should explain more precisely why they decided to use the Pittsburgh Sleep Quality Index (PSQI). It would be beneficial to discuss the strengths and weaknesses of the PSQI, with relative references, to justify its selection over other sleep quality assessment tools. This context would help readers understand the appropriateness of the PSQI for this particular study population.*

Reply: According to a review that compares and discusses sleep quality questionnaires, we have added in the Methods section: “The PSQI is the most widely used measure for assessing sleep quality, useful for epidemiological studies and easy to complete. A weakness is that different factorial structures have been found in the samples where it has been applied.”

*Similarly, the choice to use the Hopkins Symptom Checklist-25 (HSCL-25) to assess anxiety and depression symptoms requires further elaboration. The authors should provide a detailed rationale for this selection, including a discussion of the tool's strengths and weaknesses, supported by relevant references. This additional information would strengthen the methodological justification and provide a clearer picture of the assessment process.*

Reply: According to a study that evaluate the psychometric properties of the HSCL-25 in a Spanish version, we have added in Method section: “HSCL-25 is widely used for both clinical and epidemiological, self administered, easy-to-use for participants and researchers and simple of interpretation. It does not provide a clinical diagnosis; it only assesses early symptoms of anxiety and depression”.

*Moreover, in the Discussion section, the single variables considered for the association between restless legs syndrome and sleep quality in Peruvian medical students should be explained more precisely. In particular, the authors should discuss the potential influence of university location and nomophobia on the observed associations. This detailed analysis would offer deeper insights into the factors affecting the relationship between restless legs syndrome and sleep quality, thereby enhancing the discussion's comprehensiveness.*

Reply: Thank you for your commentary. In the analysis we carried out we have adjusted for both university location and nomophobia. We adjusted the statistical analysis by location since we thought it was a variable that could influence. In this way, we found that the association between restless legs syndrome was presented in the crude and multivariate analysis

*In conclusion, while the article is well written and methodologically sound, these suggested improvements would provide additional clarity and depth, thereby strengthening the manuscript further.*

**Reviewer #5.**

*Table 1, for the variable type of university, shows that the category with the highest prevalence is the private university with 50.8%. This differs from what is quoted in the item RESULTS (line spacing 3 and 4), where it is literally stated that “approximately half of them (50.8%) studied at a public university”. It is crucial to correct this interpretation to ensure an accurate understanding of the findings.*

Reply: Thanks for the comment. We have corrected the sentence.

*In the RESULTS section (lines 6 and 7), it is noted that the point estimate of poor sleep quality (22.8%) is not within the 95% confidence interval [25.9%-29.1%]. Furthermore, in Table 1, for the variable Poor Sleep Quality (PSQI), a prevalence of 77.3% is reported, which differs significantly from the 22.8% indicated both in the summary and in the results. It is recommended that the information be verified and corrected to ensure the consistency and accuracy of the data presented.*

Reply: As suggested (also by reviewer 3), we have verified and corrected the sentences in the Results section.

*In Table 1, variable “year of studies”, it is recommended that the percentages of the categories (first, second, third, fourth, fifth) be revised and adjusted so that the sum is exactly 100.0%.*

Reply: We agree. We have modified and the sum is now exact.

*In Table 1, variable “sleep quality (PSQI)”, it is recommended that the percentages of the categories (good, bad) be revised and adjusted so that the sum is exactly 100.0%.*

Reply: We have changed “77.3%” to “77.2%” in all the manuscript.

*In the interpretation of table 2 (line 5), there is a typographical error in the notation of the percentage, indicated literally as “engaged in physical activity (72.4.9%). It is recommended that it be revised as appropriate to the data being reported.*

Reply: It was actually a typographical error. We have corrected it.

*It is recommended that in the interpretation of Table 3, not only the condition of risk factor but also the protective factor that is evident for the variables physical activity where the PAR was 0.95 [0.91 - 0.99] p=0.020 from which it can be deduced that medical students who perform physical activities presented 5.0% less probability of presenting poor sleep quality, similar condition to interpret for fifth year students PAR: 0.91 IC95% (0.84 - 0.98) p=0.013.*

*Reference to calculate the percentage of probability of protection used for the variable “physical activity”:*

*0.95-1=0.05 x 100=5.0% less probability of presenting poor sleep quality.*

*5.0% (RPA=0.95, IC 95%: 0.91 - 0.99).*

Reply: In this study, we aim to assess the association between RLS and sleep quality. To better understand this relationship, we are considering potential confounding variables, including physical activity, that could influence the association. Therefore, the adjusted PR for physical activity in Table 3 is not directly interpretable.

**Reviewer #6.**

*The manuscript is clearly written and focused.I am recommending this manuscript for acceptance.*

*You need to expand your introduction/background with more information. Authors advised to recheck the language.*

Reply: We have added more information about epidemiological data and have better explained the research hypothesis, which can be seen in lines 57 to 60 and 66 to 75 of the introduction. Additionally, we have thoroughly reviewed the language.

**Reviewer #7.**

*It is an interesting topic that shows the relationship between the PSQI and IRLSSG variables and it is important to improve some points:*

*Summary: it does not specify the type and design of study.*

Reply: We have added in the abstract: “Cross-sectional study with secondary data analysis”.

*Methodology: it does not detail the research design and does not place the reliability value of the IRLSSG, the sequence of data collection is missing.*

Reply: We have specified that this is a cross-sectional study with a secondary data analysis. As suggested (also by reviewer 3), we have added information about data collection in the Methods section. Regarding the measure for restless legs syndrome, although this instrument has not undergone formal psychometric evaluation, we rely on a study that reported a sensitivity of 100% and a specificity of 88%. We have included: “This measure has a sensitivity of 100% and specificity of 88% in a Spanish version”.

*Discussion: The discussion needs to be improved; among the limitations, it should consider that the results could have been affected by some intervening variables, and a follow-up study could be carried out in this post-pandemic period.*

Reply: We have included in discussion section: “Fourth, other variables that could infuence in the association of interest, such as stress and the use of psychoactive substance, were not included”.

**Reviewer #8.**

*I welcomed the opportunity to review this manuscript. The manuscript is very brief, but fascinating; and I could read it within my scope. This study evaluated the association between RLS and sleep quality in Peruvian medical students. Findings showed that students with RLS symptoms had a 5% higher prevalence of poor sleep quality than those without RLS. Moreover, the prevalence of poor sleep quality was very high (77.3%). It must be said that this study has considerable academic value and practical significance.*

*However, I believe this manuscript leaves room for improvement, which means that the manuscript needs a certain degree of revision (if we must define the degree, it can be said to be a major revision). I will explain my opinions one by one below and request the author to respond to the review comments one by one and revise them careful.*

*Please see my review as an attachment.*

Reply: Thanks for your comment. We believe that your comments have helped improve the quality of the final manuscript.

*1. Title*

*The title clearly states the core— restless legs syndrome, sleep quality and in Peruvian medical students. However, critical time period is lacking. Most importantly, an important time period mentioned in this manuscript is the COVID-19 pandemic (lines 179–180). It has also been proven that the results of this study were greatly affected by this time period. Therefore, I think it is necessary to add “during the COVID-19 pandemic” become to “Association between restless legs syndrome and sleep quality in Peruvian medical students during the COVID-19 pandemic”.*

Reply: Thanks for your comment. Although the study was conducted during the pandemic, we cannot find arguments to support that that the association between RLS and sleep quality would differ significantly in other periods. In the discussion we mentioned that the frequency of poor sleep quality was very high (77%), but previous systematic reviews have also identified a high frequency before the pandemic (62%), as mentioned in the manuscript. However, we recognize the importance of addressing this context in the Methods section and have therefore included the information: “The primary study collected data in two stages between June 2020 and March 2021. This was done virtually through social media platforms (Facebook, WhatsApp) because universities in Peru held online classes during the pandemic to minimize the risk of contagion”.

*2. Abstract*

*The content is acceptable, there are only some formatting issues, which will be mentioned later.*

*3. Introduction*

*The introduction is very short, but it is quite informative. Generally speaking, there are the following two problems in this part:*

- *First, the review section of the Research Gap (lines 61–67) is insufficient. I think that simply saying “Previous studies have yielded heterogeneous results and have important methodological limitations, including small sample sizes and absence of control for potential confounding variables.” is not a robust gap. Here, I need you to review the situation of medical students in Peru (or at least South America) in detail and find out the real gap.*

Reply: Dear reviewer, we have followed your suggestion. We have reviewed the information gap concerning the topic of our research, detailing that sleep disorders are a very common problem among medical students. The modifications can be seen in lines 57 to 60 and 66 to 75 of the introduction.

- *Second, the research objectives should be followed by a detailed description of the research hypotheses.*

Reply: We have more clearly outlined our hypothesis on how Restless Legs Syndrome (RLS) may be associated with sleep problems. The modifications can be seen in lines 57 to 60 and 66 to 75 of the introduction.

*4. Methods*

*4.1 In (lines 74): If the content of S1 Appendix is not too long, it can be placed in the main text of the manuscript. Otherwise, not presenting this part will interfere with the reader's coherent reading flow.*

Reply: We have removed Appendix S1 and have detailed the methodology, objective, and population of the primary study. The following text was added: "The initial study was a cross-sectional analytical study aimed at identifying the factors associated with nomophobia among medical students in Peru. It was conducted in 38 medical schools and surveyed 3,139 students".

*4.2 In (line 91–125 of Variables and measures): There are a series of issues here that need to be clarified or revised by the author:*

- *If I counted correctly, there are four established scales used here, namely Pittsburgh Sleep Quality Index (PSQI), International Restless Legs Syndrome Study Group (IRLSSG), Hopkins Symptom Checklist-25 (HSCL-25) and Nomophobia Questionnaire (NMP-Q). All the Cronbach’s alpha coefficients in these four scales are not from your current research, but are cited and summarized from previous research. This practice can be called “reliability induction” in psychometrics, please refer to the two journal articles in brackets (https://doi.org/10.1177/0013164403261814; https://doi.org/10.1177/00131640021970682). This is an inappropriate approach. Therefore, I ask you to report the Cronbach’s alpha and McDonald’s omega coefficients for each dimension you are currently studying.*

Reply: We have calculated Cronbach's alpha for each measure and specified it in the Methods section. For PSQI we added: “In our sample, the Cronbach's alpha was 0.75”. For HSCL-25 we added: “For our sample, Cronbach's alpha was 0.90 for anxiety and 0.93 for depression”. For Nomophobia Questionnaire, we added: In our sample, Cronbach’s alpha was 0.88 for the factor "not being able to access information", 0.87 for "giving up convenience", 0.95 for "not being able to communicate", and 0.93 for "loss of connection".

However, the instrument for restless legs syndrome is a set of three questions based on the criteria for detecting restless legs by the International Restless Legs Syndrome Study Group (IRLSSG). This measure does not have the evaluation of psychometric properties, but we are based on a study in which a sensitivity of 100% and specificity of 88% was found. We have included: “This measure has a sensitivity of 100% and specificity of 88% in a Spanish version”.

- *I checked the journals you cited regarding the Spanish versions of the instruments. Yes, they all show that the psychometric properties of the Spanish versions of the instruments are adequate. However, you did not specify where you obtained the instruments. Therefore, the journals you cited do not always have an appendix showing the official Spanish version of the instruments. I hope you explain how you obtained the instruments; these are requirements for Open Science. The following is an example for your reference: “The SDQ is a brief screening instrument to measure behavioral difficulties, capturing the MHS of children and adolescents aged 4–16 years [?]. It comprises 25 items across five dimensions: Emotional Symptoms, Conduct Problems, Hyperactivity/Inattention, Peer Problems, and Prosocial Behavior, with five items in each dimension. Responses are categorized into a 3-point scale (0 = not true, 1 = somewhat true, 2 = certainly true), with a six-month recall period. A few items are reverse-scored. After recoding reverse-scored items, the total difficulties score (TDS) is obtained by summing up scores from all 20 items except those in the Prosocial Behavior. The possible range of TDS is 0–40, with cutoff values from the Shanghai norms classifying it as “Normal” (0–14), “Borderline” (15–17), and “Abnormal” (18–40) [?]. The Mandarin Chinese self-reported SDQ used in this study has been validated previously [??], and it was excerpted from the “Handbook of Commonly Used Psychological Assessment Scales” published in the Chinese Mainland [?]. Its internal consistency of the TDS was appropriate, with Cronbach’s alpha (α) coefficient of 0.936 and McDonald’s omegas (ωt) coefficient of 0.943.”*

Reply: We would like to clarify the following: for the PSQI sleep quality instrument, the Spanish version is available in the appendix of the reference we provided. For the restless legs syndrome and nomophobia instruments, the Spanish versions are included within the cited study. For the anxiety and depression instrument, we cited a validated Spanish version; however, the authors of that study did not include the items in Spanish, so we also referenced a document with the Spanish version translated by the Peruvian Ministry of Health. We have specified this in the manuscript.

- *I noticed that you only introduced the scoring criteria for Hopkins Symptom Checklist-25 (HSCL-25) and Nomophobia Questionnaire (NMP-Q), but did not introduce how to use them to divide the test subjects into Yes/No. However, your Table 1 does show the division, and the Note of Table 1 does not indicate the division criteria. Therefore, I think you need to add the division criteria when introducing the instruments.*

Reply: We agree. We have added information in the Measures section for HSCL-25: “If participants has an average score above 1.75 on the anxiety or depression subscales of the HSCL-25, they are considered to have possible symptoms of anxiety or depression, respectively”. Also for NMP-Q: “The following cut-off points were applied for interpreting the NMP-Q questionnaire: absense (a score of 20), mild (greater than 20 but less than 60), moderate (a score of 60 or higher but less than 100) and severe (a score of 100 or higher). A systematic review found that the NMP-Q demonstrates excellent internal consistency, with strong structural validity across its four-factor model”.

- *If you follow the Open Science requirements, your appendix should include the complete questionnaire (including four established scales) you used in your current study. Please consider adding it. If you cannot add it, please explain why.*

Reply: As suggested (also by reviewer 2) we have added the questionnaire as supplementary material (Supplementary material 1).

*5. Results*

*There are some formatting issues in the Results section, which require review and revision by the authors.*

*5.1 In (Table 1–3): Please ensure that your manuscript contains only English, unless there are special cases. For example, if there is a Spanish “18 a 20” “21 a 23” “24 a más” in Table 1–3, please correct it to English.*

Reply: We appreciate the detail of your observation, and we have made the language corrections.

*5.2 In (Table 2):*

- *If I’m not mistaken, most of the data in Table 2 may use chi-square tests, but you didn’t indicate it and only showed the p-values. Here, I need you to add the data of the corresponding test statistics. For example, if you use chi-square tests, report χ². In addition, report the effect size of each test.*

Reply: Dear reviewer, indeed, the test used was the chi-square test, which is detailed in the footnote of the table. The superscript asterisk next to the P-value indicates that all mentioned P-values were obtained using this test.

- *The bottom line of Table 2 is missing, please add it.*

Reply: The missing line was added to Table 2.

*5.3 In (entire “5. Results” section and entire manuscript, including references):*

*You have used hyphen (-) in all Table, References and entire manuscript. Is this appropriate? Please refer to the relevant information to distinguish the difference between these four symbols and the corresponding application:*

- *Hyphen: -*
- *Minus sign (negative sign): −*
- *EN dash: –*
- *EM dash: —*

Reply: Dear reviewer, we appreciate the detail of your observation. We have determined that the EM dash symbol '—' is the most appropriate for Table 3. We have added its meaning in the footer.

*6. Discussion*

*In (lines 212–215): Enter 2014 first and then press Alt + X “On the other hand, this study is the first to evaluate the relationship between restless legs syndrome and sleep quality in medical students, using a multivariable model in which other confounding variables were adjusted and with a larger sample size than used in previous research.” This section obviously states the strengths of the research. However, it is not logical to put it here. You should put this section in the first paragraph of the discussion. Because the first paragraph of the discussion usually introduces the overall situation of the research and its uniqueness.*

Reply: We followed your suggestion, removed the strengths from the limitations section, and placed them at the beginning of the discussion as follows “This is the first study to evaluate the relationship between restless legs syndrome and sleep quality among medical students, with a larger sample size than that used in previous research.”

*7. References*

*Accurate referencing is a key component of good academic practice and enhances the presentation of your work: it shows that your writing is based on knowledge and informed by appropriate academic reading. The Reference format of the PLOS ONE is the AMA Style. However, you did not present the literature strictly as required by the AMA Style. You can refer to the AMA Manual of Style (11th edition). Please pay attention to the following problems, and step by step, manually correct these issues. Please do not only use bibliographic management software (because if there is information missing in the original document, the data exported by the bibliographic management software will also be wrong). In addition, each of your references lacks doi. Please refer to the format in brackets (https://doi.org/10.1111/j.1524-4733.2007.00293.x) to add it and add it as a hyperlink.*

Reply: We followed your suggestions and have modified and corrected our references.

**Reviewer #9.**

*Dear author,*

*In this study, you investigated the relationship between restless legs syndrome and sleep quality among medical students. Although this topic has been investigated before, the high number of participants in your study is noteworthy. The manuscript is written in great detail. However, it needs minor revisions, which I have outlined below.*

*- The language of the manuscript should be revised.*

*- Spelling mistakes should be corrected.*

*- Page 13, Line 139-40. The information here does not match the table.*

*Best wishes*

Reply: We appreciate the comments and have made changes to the manuscript. Regarding line 139-140, we have made the corrections, as recommended (also by reviewer 3).

**Reviewer #10.**

*Dear authors: Epidemiological data are always required in the introduction. Furthermore, a non-probabilistic sample was chosen, which does not guarantee that these results are valid for the general population and, finally, there is no biological plausibility to explain the relationship between both variables.*

- Reply:
- Dear reviewer, we have added epidemiological information in the introduction following your suggestion.
- It is true that the results may not be valid for the entire general population; this limitation is detailed in the relevant section.
- Regarding biological plausibility, there are studies that show this possible relationship, detailing that the increase in involuntary leg movements during sleep could affect the circadian cycle. It is not entirely clear if this is the pathway through which Restless Legs Syndrome (RLS) could be associated with sleep disorders; this is also detailed in the introduction section, lines 57 to 60 and 66 to 75.
