# Supplementary material for: Association between restless legs syndrome and sleep quality in Peruvian medical students
Source: PLoS One. 2025 Mar 25;20(3):e0320008. doi: 10.1371/journal.pone.0320008 (PMC11936163; doi:10.1371/journal.pone.0320008)
Supplement: Attachment — Submitted filename: Response to Reviewers 2.docx [file pone.0320008.s006.docx]

Dear editor,

Many thanks for your response to the submission of our paper

Our detailed responses to each of their points are provided below, with the original comments presented in italics.

*1. It is recommended that the model fitting methodology be based on literature references that clearly state that the adjustment or confounding variables are not directly interpretable, allowing the interpretation to focus exclusively on the independent variable or variable of interest.*

*I suggest that once the relevant references have been cited, a clear specification of the independent variable, the dependent variable and the adjustment or confounding variables should be included in the methodology section. In addition, it would be advisable to add a footnote to the relevant table indicating that certain variables are not interpretable and giving a brief justification. This will provide greater clarity for the reader and reduce the risk of misinterpretation of the role of the adjustment variables.*

*You may find it useful to consult the following reference:*

*Akinkugbe AA, Simon AM, Brody ER. A scoping review of the Table 2 fallacy in the oral health literature. Community Dent Oral Epidemiol. 2021 Apr;49(2):103-109. doi: 10.1111/cdoe.12617.*

**Reply:** We have added the following in Data analysis section:
“It is essential to emphasize that only the estimate of the association between RLS and sleep quality should be interpreted, while the estimates for the confounding variables should not be considered[^30^](https://www.zotero.org/google-docs/?9i0SbH)”.

We also included in Table 3:

“**Only this adjusted RP should be interpreted, the rest of adjusted RP correspond to the confounding variables so they should not be interpreted”.

Finally, we included in Variables section which was the independent, dependent and confounding variables.

*2. A Poisson regression model with robust variance was used in the multivariate analysis. It is recommended that the authors justify the choice of this model instead of logistic regression, as this explanation provides important information to the reader. The following reference may be helpful: Thompson ML, Myers JE, Kriebel D. Prevalence odds ratio or prevalence ratio in the analysis of cross-sectional data: what to do? Occup Environ Med. 1998 Apr;55(4):272-7. doi: 10.1136/oem.55.4.272*

**Reply:** We used this regression model since the frequency of inadequate sleep quality was high so using logistic regression (i.e., calculating odds ratios) could overestimate the objective association[^29^](https://www.zotero.org/google-docs/?GIKbck)
